# Supplementary material for: Global genetic rewiring during compensatory evolution in the yeast polarity network
Source: EMBO Rep. 2026 Feb 16;27(6):1414–36. doi: 10.1038/s44319-026-00709-4 (PMC13022240; doi:10.1038/s44319-026-00709-4)
Supplement: Supplementary file 1 — Appendix [file 44319_2026_709_MOESM1_ESM.pdf]

Appendix

Contents

A Read count correction and normalization procedure . . . 1
B Verification of background-dependent fitness effects of *AXL2* and *DYN1* deletions . . . 2
C The possible effects of genetic markers on SATAY . . . 2
D Appendix Figures . . . 4
S1. Normalization procedure increases correlation in the summed read counts per gene between replicate experiments . . . 4
S2. Images of the transformation plates after transformation with an *axl2*Δ construct and a *dyn1*Δ construct in the wild-type strain the polarity mutant . . . 5
S3. Illustration of the procedure used to determine colony numbers and size from images of the transformation plates . . . 6
S4. Comparison of the colony numbers and sizes after the deletion of *AXL2* and *DYN1* in the wild-type strain and the polarity mutant . . . 7
S5. Effects of the *HIS3* auxotrophic marker on the SATAY screen . . . 8
S6. Effects of mating type differences on the SATAY screen . . . 9

A. Read count correction and normalization procedure. The benefit of including read counts in our metric to identify changes in the fitness effect of gene disruptions across genetic backgrounds is that it allows for a more sensitive analysis than those based on insertion counts. For example, a gene may have a negligible difference in insertion counts between two genetic backgrounds if the change in gene disruption tolerance is not so drastic that it causes a large number of mutants to fall below the detection level in one of the two backgrounds. However, if a significant difference in gene disruption tolerance truly exists, we would expect that the read counts associated with each insertion site (which reflect mutant abundance) will be lower in one of the two strains. Thus, extending the analysis from a binary assessment of the occupancy of insertion sites to a gradual scale based on read counts can uncover differences that would otherwise remain undetected. However, a drawback is that read counts tend to be more variable between replicate experiments than insertion counts, often owing to read count spikes caused by technical and biological artifacts (DeJesus et al. 2016). This increased noise level can hinder the desired increase in sensitivity of the analysis. In addition, spikes in the read counts can significantly skew the read count distribution. This means that the small subset of insertion sites with an artificially high read count will strongly affect methods that aim to correct differences in sequencing depth between libraries by a linear transformation of the total read count.

To address these issues, we implemented the Beta-Geometric correction (BGC) method developed by DeJesus et al. (2013). This method is based on the observation that read count distributions obtained from transposon insertion sequencing (TIS) experiments resemble a geometric distribution where most insertion sites have a low read count, while only few sites contain many reads. During the BGC correction procedure, the quantiles of the empirical read count distribution are adjusted to match the quantiles of a fitted 'ideal' geometric distribution. To allow for greater flexibility of the fit, this ideal distribution is implemented as a mixture of geometric distributions with a Beta prior on the probability parameter. In practice, this procedure corrects for the 'skew' in the empirical read count distribution by suppressing spikes while inflating the insertion sites that have a low read count. The results in Appendix Figure S1 show that the application of this procedure greatly improves the correlation between replicate datasets (Appendix Figure S1b) compared to uncorrected datasets (Appendix Figure S1a). Lastly, after applying the BGC correction, we normalized for differences in sequencing depth and library complexity across datasets using the median of ratios normalization (Anders et al. 2010; Robinson et al. 2010). In summary, the geometric mean across all samples was calculated for each gene:

$$\bar{X}_g = \exp \frac{1}{n} \cdot \sum_{a}^{n} \ln\left(R_{g,a}\right)). \qquad [1]$$

Where $\bar{X}_g$ is the geometric mean of the read counts mapping to gene g, n is the total number of datasets and $R_{g,a}$ is the number of reads that map to gene g in dataset a. Next, the ratio of the total read count to the geometric mean $\bar{X}_g$ was determined for each sample and the sample-specific normalization factor was taken to be the median of these ratios across all genes:

$$S_a = \text{median}(\frac{R_{g,a}}{\bar{X}_g}) \ \text{, for all } a \in G \tag{2}$$

With S_a the normalization factor for dataset a and G is the complete set of annotated genes used in this chapter. Finally, the read counts were linearly scaled by the normalization factor S_a:

$$R_{g,a,\text{norm}} = \frac{R_{g,a}}{S_a}. \tag{3}$$

B. Verification of background-dependent fitness effects of *AXL2* and *DYN1* deletions. We validated the differential functional importance of two genes identified in our SATAY screen, *AXL2* and *DYN1*, by deleting them in both the wild-type (replicate yEK19a) and polarity mutant (replicate yEK23a) backgrounds. *AXL2* and *DYN1* were chosen because they represent one case where the disruption tolerance of the gene decreases in the polarity mutant (*AXL2*, $\log_2$(FC) = -2.8) and one case where the disruption tolerance increases (*DYN1*, $\log_2$(FC) = 2.1 relative to the wild-type (Appendix Figure S4a). We quantified fitness differences arising from gene deletions across the two genetic backgrounds by scoring colony number and size directly from the transformation plates. This approach is based on the assumption that variation in colony number reflects differences in survival rate, whereas variation in colony size reflects differences in growth rate. To assess reproducibility, transformations were performed in triplicate, and replicate plates yielded similar colony densities and sizes (see Appendix Figure S2). For both *AXL2* and *DYN1*, we find that the median number of colonies on the transformation plate is lower in the polarity mutant (figures S4b and e), while the median colony size is lower for the wild-type strain (Appendix Figure S4c and f). When considering the total area covered by colonies across the three replicate plates, colonies from the *AXL2* deletion in the polarity mutant covered slightly less area than in the wild-type strain (Appendix Figure S4d). In contrast, the *DYN1* deletion resulted in a greater total colony-covered area in the polarity mutant than in the wild-type (Appendix Figure S4g). Thus, although deletion of *AXL2* and *DYN1* both reduce colony number and increase colony size in the polarity mutant compared with the wild-type, the combined effect these two metrics leads to distinct overall cell yields for the two genes.

While it is difficult to predict how colony number and colony size combine into an overall strain fitness, these results suggest that the fitness effects caused by deleting *AXL2* and *DYN1* differ between the polarity mutant and the wild-type strain. Nonetheless, we recognize that these data are preliminary. For example, differences in tolerance to the heat shock used during transformation, as well as the emergence of suppressor mutations, could have influenced these results. Therefore, additional experiments will be required to confirm these findings.

C. The possible effects of genetic markers on SATAY. The polarity mutant used in this study was constructed based on mutations that were identified to compensate for the loss of Bem1 after experimental evolution (Laan et al. 2015). Although using a reconstructed strain offers the advantage of being free from potentially undiscovered or hitchhiker mutations (Buskirk et al. 2017), the genetic elements introduced into the strain during construction may affect the fitness landscape. This section outlines the key genetic differences between the polarity mutant and the wild-type strain and discusses their potential impact on the outcomes of our genome-wide screen.

First, the polarity mutant was constructed by replacing *BEM1*, *BEM3*, and *NRP1* with the kanMX, NatMX, and hphMX cassettes, respectively. These cassettes allow the selection of the desired genotype following transformation by conferring resistance to specific drugs. Studies have demonstrated that the expression of drug resistance cassettes does not significantly affect cell growth when integrated at a neutral locus (Baganz et al. 1997; Goldstein et al. 1999), suggesting that these markers do not affect the native cellular physiology. Further evidence that drug markers do not significantly impact the fitness landscape comes from genome-wide screens using the Synthetic Genetic Array (Tong, Evangelista, et al. 2001). These screens have successfully uncovered a significant portion of the cell's functional wiring by making gene deletions by using the same drug resistance cassettes to generate gene deletion mutant arrays (Boone et al. 2007; Tong, Lesage, et al. 2004). If these selection markers had a dominant influence on the fitness landscape, we expect such effects would leave a characteristic signature in these screens.

Second, the polarity mutant and the wild-type strain differ at their *CAN1* locus. Although both strains carry an inactive version of the *CAN1* gene, the mutant strain has this gene replaced by the MFA1pr–HIS3 construct (commonly known as the magic marker (Boone et al. 2007; Singh et al. 2009)) whereas in the wild-type strain, the locus is inactivated through the introduction of an early stop codon (Maderazo et al. 2000). The use of auxotrophic markers during strain construction is known to have a considerably greater impact on cellular physiology than the use

of drug markers (Baganz et al. 1997; Alam et al. 2016; Kaplan et al. 2024). For example, auxotrophic markers have been shown to alter the drug sensitivity of yeast strains (Kaplan et al. 2024), alter their growth pattern compared to the prototrophic strain (Mülleder et al. 2012) and interact epistatically with other auxotrophic markers (Alam et al. 2016). For histidine, it has been demonstrated that converting an auxotrophic strain into a prototroph through the introduction of the *HIS3* marker results in at least two-fold change in the expression of 15 metabolic genes. Despite this change, the expression profile of the histidine prototrophic strain remained relatively similar to its auxotrophic ancestor when compared to the effect of other auxotrophic markers (Alam et al. 2016). Thus, the use of the *HIS3* marker in our polarity mutant could have affected the results of our screen. To gain more insights into the possible consequences of using the *HIS3* marker, we checked whether our set of significantly different genes was enriched for its genetic interactors (Appendix Figure S5. The genetic interactions of *HIS3* were retrieved from the SGD database (accessed on 15/02/2025). We found that from the 34 interacting genes listed on SGD, 6 show a significant difference in disruption tolerance between our polarity mutant and wild-type strains (Appendix Figure S5a), meaning that our dataset is not enriched for genetic interactors of *HIS3* (p-value = 0.4, Fisher's exact test). Next, we checked whether we could see the effect of the difference in histidine auxotrophy between the two strains based on the other genes in the histidine biosynthesis pathway (GO term GO:0000105). Indeed, 6 of 7 of the genes involved in histidine synthesis (*HIS1* was the only exception) showed up as significant in our screen (Appendix Figure S5b). All these genes have a $\log_2(FC)<0$ (Appendix Figure S5b), indicating that their disruption incurs a greater fitness defect in the prototrophic polarity mutant than in the auxotrophic wild-type strain. This result aligns with observations from other studies demonstrating that prototrophy generally confers a growth advantage that cannot be replicated in auxotrophic strains, even with nutrient supplementation in the growth medium (Alam et al. 2016; Mülleder et al. 2012). Further evidence supporting this idea is our observation that in the polarity mutant, genes associated with amino acid transport (GO term GO:0006865) exhibit greater tolerance to disruptions compared to those in the wild type, with the exception of *GNP1* (Appendix Figure S5c). Why *GNP1*, a high-affinity glutamine permease, deviates from this trend is unclear. Overall, the observed changes in disruption tolerance for genes involved in histidine synthesis and import align with the expected effects of introducing *HIS3*. Consequently, there are no indications that the introduction of this marker has served as a mediator for genetic rewiring in the polarity mutant.

Lastly, the polarity mutant is of mating type **a** while the wild-type strain is of mating type α. The two mating types are genetically indistinguishable, with the exception of 13 mating type-specific genes (8 MAT**a** and 5 MATα specific genes) (Galgoczy et al. 2004) and the conformation of chromosome III (Belton et al. 2015). Although several mating type-specific genes are present in our set (Appendix Figure S6a), they were generally less tolerant to disruptions in the polarity mutant than in the wild-type, regardless of whether they are MAT**a** or MATα specific. Moreover, despite the conformational differences of chromosome III, which could potentially influence its accessibility to transposons, the number of differentially tolerant genes on chromosome III was not found to differ substantially compared to other chromosomes (Appendix Figure S6b).

In summary, we have assessed the effects of the genetic differences between our polarity mutant and the wild-type strain (in addition to the deletions of *BEM1*, *BEM3*, and *NRP1*) that were introduced during strain construction. SATAY is a robust and sensitive screening method capable of detecting differences caused by genetic elements often presumed inert during strain construction. This sensitivity is also visible in our dataset, mainly by the impact of the *HIS3* marker on amino acid synthesis and import. The impact of *HIS3* on the metabolic state may explain the presence of a cluster enriched for metabolic genes in our set of differentially tolerant genes (figure EV4). Consequently, we cannot rule out the possibility that the markers or mating-type differences introduced during strain construction influenced the observed global genetic rewiring. For example, the *HIS3* marker could potentially interact with the *bem1*Δ*bem3*Δ*nrp1*Δ mutation and thereby have a significant effect on the difference in the fitness landscape between the polarity mutant and wild-type strain. However, such a scenario appears unlikely, given the remarkably predictable changes in disruption tolerance among genes involved in histidine synthesis and amino acid transport. Furthermore, even if this were the case, our findings still demonstrate that constructing a mutant that polarizes almost as efficiently as the wild-type strain despite lacking a key polarity gene drastically alters the fitness landscape.

D. Appendix Figures.

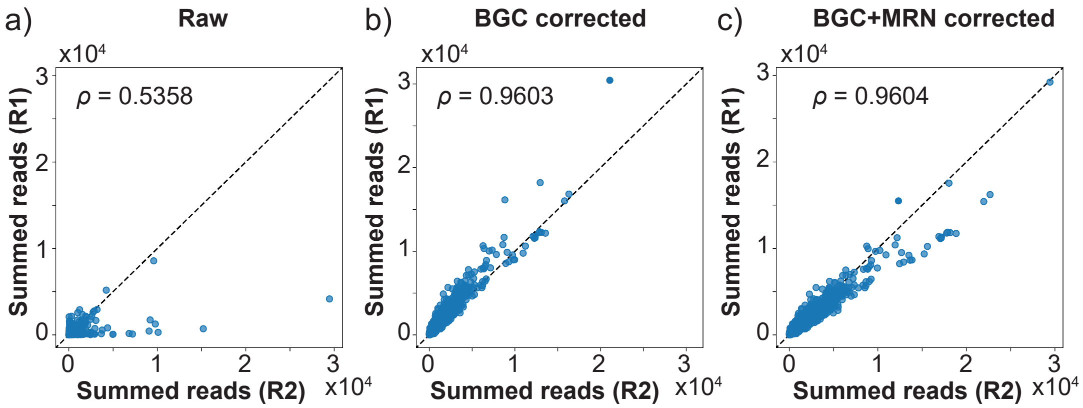

Appendix Figure S1. Normalization procedure increases correlation in the summed read counts per gene between replicate experiments. In order to improve the correlation between replicate datasets derived from the same genetic background, the datasets are first aligned with their best fitting Beta-Geometric distribution (panel (a) to panel (b)) to correct for spikes in read counts, followed by a median of ratios normalization to account for differences in sequencing depth (panel (b) to panel (c)). In each panel, the value of Pearson's correlation coefficient (ρ) is shown.

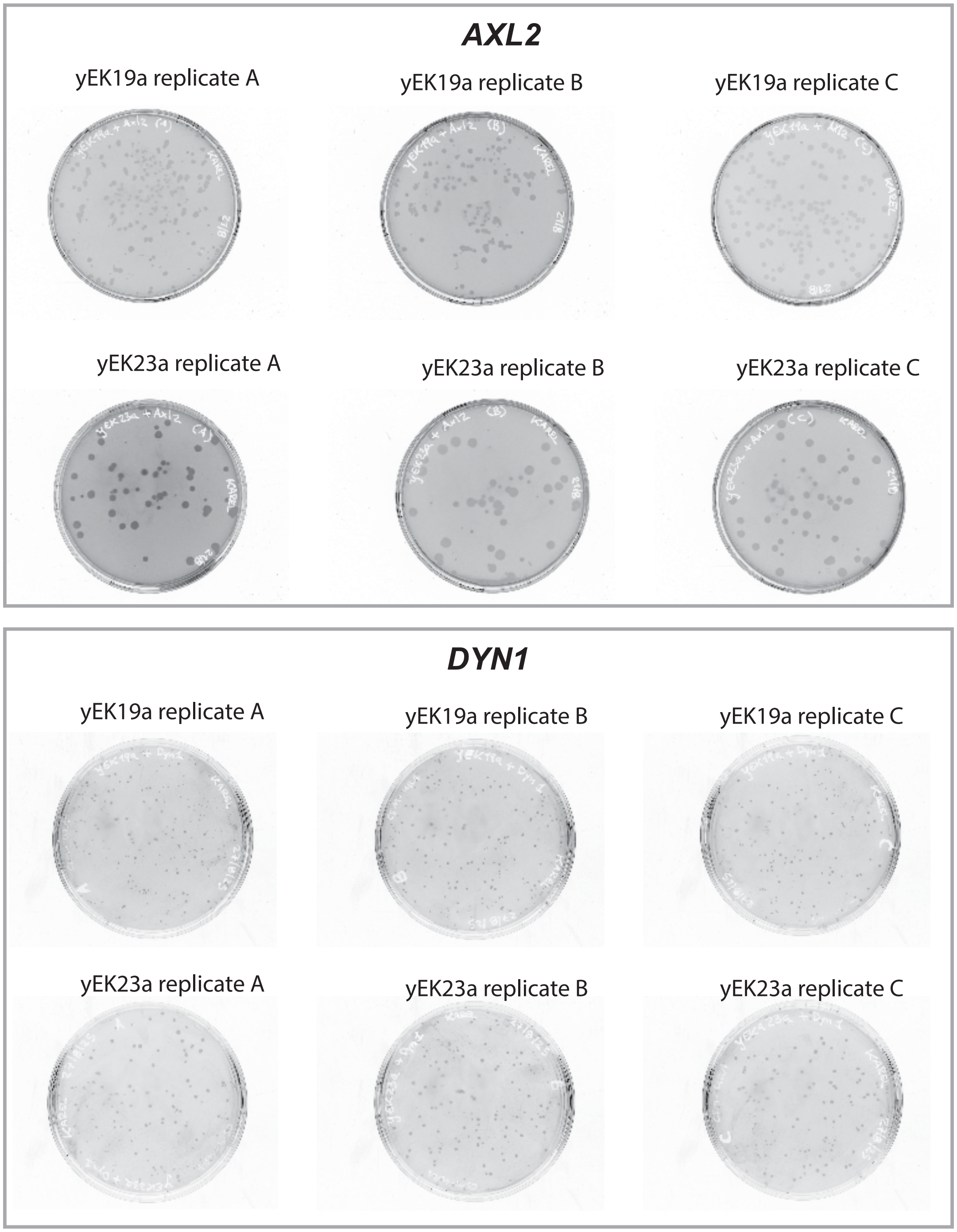

Appendix Figure S2. Images of the transformation plates after transformation with an *axl2*△ construct (top) and a *dyn1*△ construct (bottom) in the wild-type strain (yEK19a) and the polarity mutant (yEK23a). Three replicate transformations (A-C) were performed for each genetic background to assess the reproducibility of the transformation.

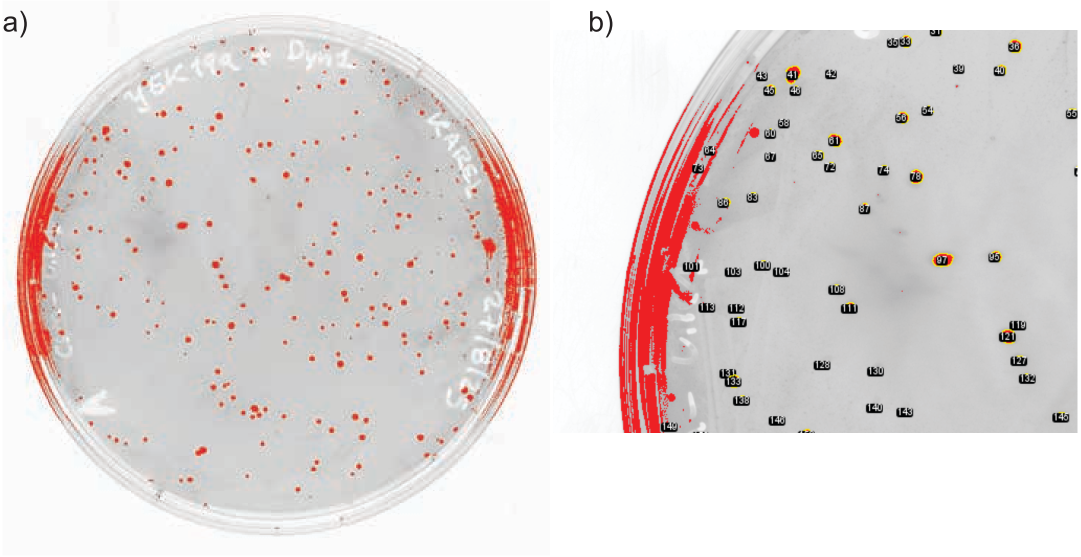

Appendix Figure S3. Illustration of the procedure using to determine colony numbers and size from images of the transformation plates. (**a**) Transformation plate of yEK19a carrying the *DYN1* construct after application of a pixel-intensity threshold used for binarization. Pixels within the chosen lower and upper limits of the threshold are highlighted in red. (**b**) Zoom-in of the plate edge shown in (a). Areas outlined in yellow and numbered are colonies identified by ImageJ's Analyze Particles function. The image shows that this procedure can correctly identify colonies while mitigating noise caused by reflections from the plate edges.

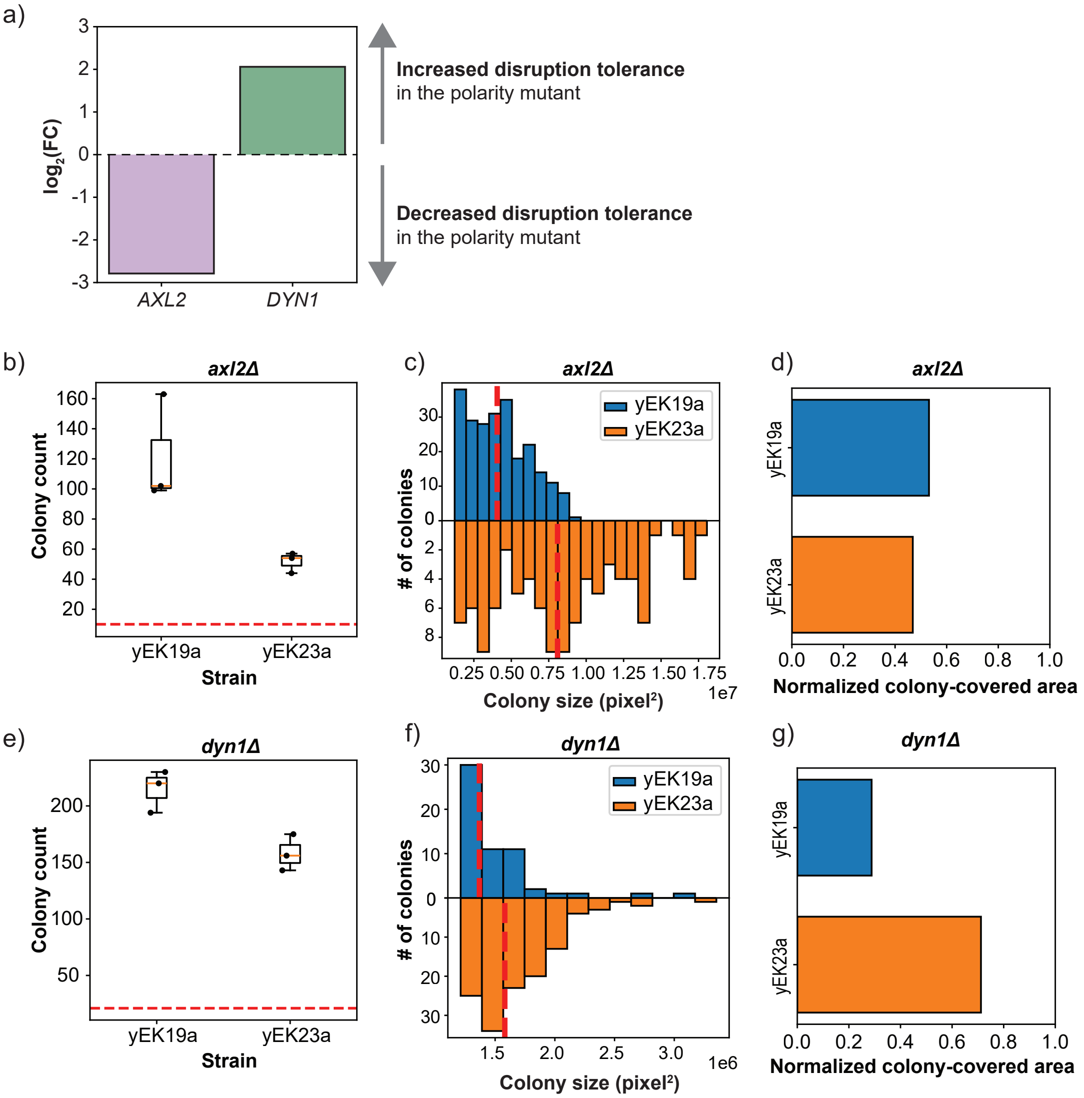

Appendix Figure S4. Comparison of the colony numbers and sizes after the deletion of *AXL2* and *DYN1* in the wild-type strain (yEK19a) and the polarity mutant (yEK23a). (**a**) Log-fold changes in transposon insertion density for *AXL2* and *DYN1* that were determined with the SATAY screen. *AXL2* exhibits a reduced tolerance to transposon disruptions in the polarity mutant relative to the wild-type strain, indicating that it becomes less dispensable in the polarity mutant background. In contrast, *DYN1* shows an increased tolerance to transposon disruptions in the polarity mutant, suggesting that it becomes more dispensable. (**b**) The colony counts of the transformation plates from yEK19a and yEK23a after transformation with an axl2delta construct. The deletion of *AXL2* resulted in a reduced colony count in strain yEK23a compared to yEK19a. In the boxplots, the solid orange lines indicate the median values, while the boxes represent the interquartile range (IQR). The whiskers extend to the most extreme data points within 1.5 times the IQR. Each black dot corresponds to the colony count from an individual transformation plate. The dashed red line represents the average colony count from the negative control plates and denotes the expected background count caused by imperfections in the colony segmentation procedure. (**c**) The colony size distributions of transformation plates for yEK19a (blue) and yEK23a (orange). The dashed red lines represent the median values. Only colonies with an area exceeding the largest colony size obtained from the negative control plates are included in the distributions. Following the *AXL2* deletion, colonies of yEK23a were generally larger than those of yEK19a. (**d**) The total colony-covered area for transformation plates of yEK19a and yEK23a after the deletion of *AXL2*. Normalized values were obtained by summing the sizes of all identified colonies across three replicate plates and dividing by the combined total colony-covered area of both strains. In the *axl2*Δ background, the colony-covered area was slightly smaller for yEK23a than for yEK19a, suggesting that the plates from yEK23a contain fewer cells. (**e-g**) The same visualizations as in (**b-d**), but for the deletion of *DYN1*. The colony counts (**e**) and colony size distributions (**f**) of the *dyn1*Δ transformation plates show the same pattern as for the *axl2*Δ transformation plates. In contrast, the normalized colony-covered area (**g**) is greater for yEK23a than for yEK19a in the *dyn1*Δ background.

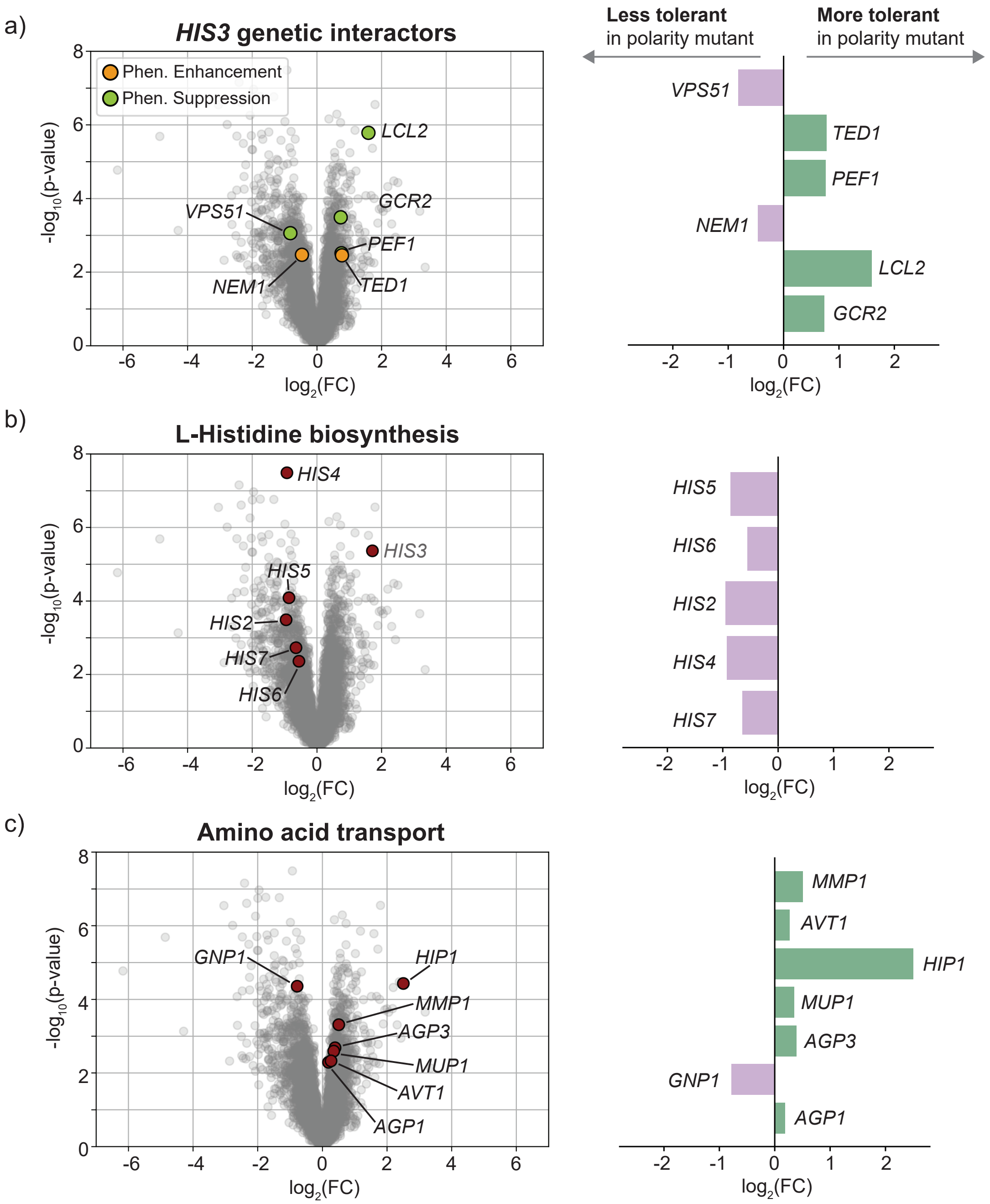

Appendix Figure S5. Effects of the *HIS3* auxotrophic marker on the SATAY screen. Volcano plots highlighting: (**a**) genetic interactors of *HIS3*, (**b**) genes involved in L-histidine biosynthesis, and (**c**) genes involved in amino acid transport. Only genes with an adjusted p-value (p_{adj}) below 0.05 are annotated. The panel to the right of each volcano plot provides an overview of the log-fold changes in disruption tolerance for each annotated gene.

a)

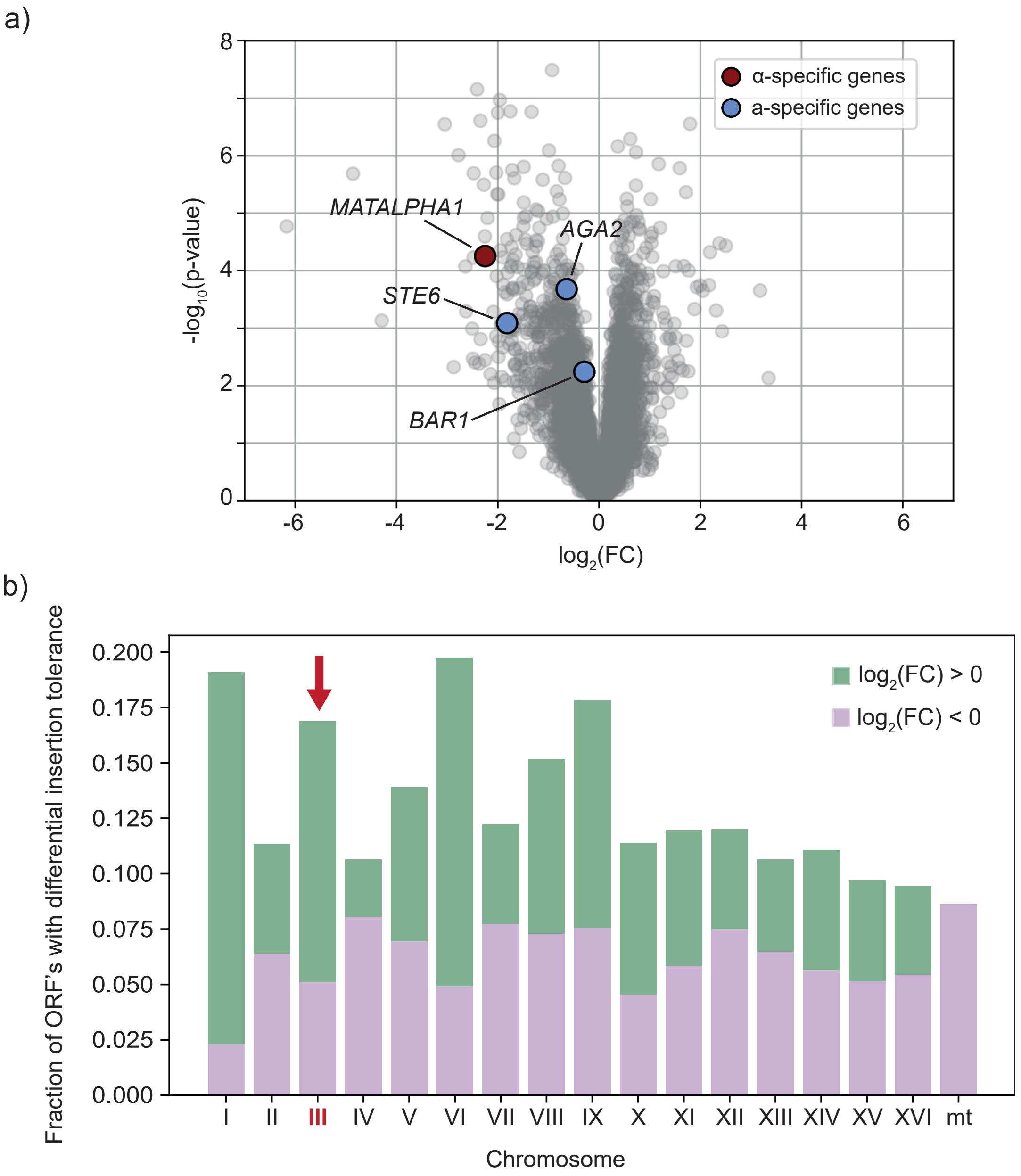

Appendix Figure S6. Effects of mating type differences on the SATAY screen. (**a**) Volcano plot in which α- and a-specific genes are annotated. Only genes with Only genes with an adjusted p-value (p_{adj}) below 0.05 are annotated. (**b**) The fraction of annotated genes on each chromosome identified as differentially tolerant to transposon insertions in our SATAY screen. Chromosome III, which has a different conformation in the two mating types, is annotated by the red arrow.

References

Alam M. T., Zelezniak A., Mülleder M., Shliaha P., Schwarz R., Capuano F., Vowinckel J., Radmaneshfar E., Krüger A., Calvani E., et al. (2016). The metabolic background is a global player in Saccharomyces gene expression epistasis. *Nature Microbiology* 1: 15030.

Anders S. and Huber W. (2010). Differential expression analysis for sequence count data. *Genome Biology* 11: R106.

Baganz F., Hayes A., Marren D., Gardner D. C. J., and Oliver S. G. (1997). Suitability of replacement markers for functional analysis studies inSaccharomyces cerevisiae. *Yeast* 13: 1563–1573.

Belton J.-M., Lajoie B. R., Audibert S., Cantaloube S., Lassadi I., Goiffon I., Baù D., Marti-Renom M. A., Bystricky K., and Dekker J. (2015). The Conformation of Yeast Chromosome III Is Mating Type Dependent and Controlled by the Recombination Enhancer. *Cell Reports* 13: 1855–1867.

Boone C., Bussey H., and Andrews B. J. (2007). *Exploring genetic interactions and networks with yeast.*

Buskirk S. W., Peace R. E., and Lang G. I. (2017). Hitchhiking and epistasis give rise to cohort dynamics in adapting populations. *Proceedings of the National Academy of Sciences* 114: 8330–8335.

DeJesus M. A. and Ioerger T. R. (2013). A Hidden Markov Model for identifying essential and growth-defect regions in bacterial genomes from transposon insertion sequencing data. *BMC Bioinformatics* 14: 303.

– (2016). Normalization of transposon-mutant library sequencing datasets to improve identification of conditionally essential genes. *Journal of Bioinformatics and Computational Biology* 14: 1642004.

Galgoczy D. J., Cassidy-Stone A., Llinás M., O'Rourke S. M., Herskowitz I., DeRisi J. L., and Johnson A. D. (2004). Genomic dissection of the cell-type-specification circuit in Saccharomyces cerevisiae. *Proceedings of the National Academy of Sciences* 101: 18069–18074.

Goldstein A. L. and McCusker J. H. (1999). Three new dominant drug resistance cassettes for gene disruption inSaccharomyces cerevisiae. *Yeast* 15: 1541–1553.

Kaplan K., Levkovich S. A., DeRowe Y., Gazit E., and Laor Bar-Yosef D. (2024). Mind your marker: the effect of common auxotrophic markers on complex traits in yeast. *The FEBS Journal* 291: 2209–2220.

Laan L., Koschwanez J. H., and Murray A. W. (2015). Evolutionary adaptation after crippling cell polarization follows reproducible trajectories. *eLife* 4.

Maderazo A. B., He F., Mangus D. A., and Jacobson A. (2000). Upf1p Control of Nonsense mRNA Translation Is Regulated by Nmd2p and Upf3p. *Molecular and Cellular Biology* 20: 4591–4603.

Mülleder M., Capuano F., Pir P., Christen S., Sauer U., Oliver S. G., and Ralser M. (2012). A prototrophic deletion mutant collection for yeast metabolomics and systems biology. *Nature Biotechnology* 30: 1176–1178.

Robinson M. D. and Oshlack A. (2010). A scaling normalization method for differential expression analysis of RNA-seq data. *Genome Biology* 11: R25.

Singh I., Pass R., Togay S. O., Rodgers J. W., and Hartman J. L. (2009). Stringent Mating-Type-Regulated Auxotrophy Increases the Accuracy of Systematic Genetic Interaction Screens with Saccharomyces cerevisiae Mutant Arrays. *Genetics* 181: 289–300.

Tong A. H. Y., Evangelista M., Parsons A. B., Xu H., Bader G. D., Pagé N., Robinson M., Raghibizadeh S., Hogue C. W. V., Bussey H., et al. (2001). Systematic Genetic Analysis with Ordered Arrays of Yeast Deletion Mutants. *Science* 294: 2364–2368.

Tong A. H. Y., Lesage G., Bader G. D., Ding H., Xu H., Xin X., Young J., Berriz G. F., Brost R. L., Chang M., et al. (2004). Global Mapping of the Yeast Genetic Interaction Network. *Science* 303: 808–813.
