# Supplementary material for: Global genetic rewiring during compensatory evolution in the yeast polarity network
Source: EMBO Rep. 2026 Feb 16;27(6):1414–36. doi: 10.1038/s44319-026-00709-4 (PMC13022240; doi:10.1038/s44319-026-00709-4)
Supplement: Supplementary file 8 — Expanded View Figures [file 44319_2026_709_MOESM8_ESM.pdf]

Expanded View Figures

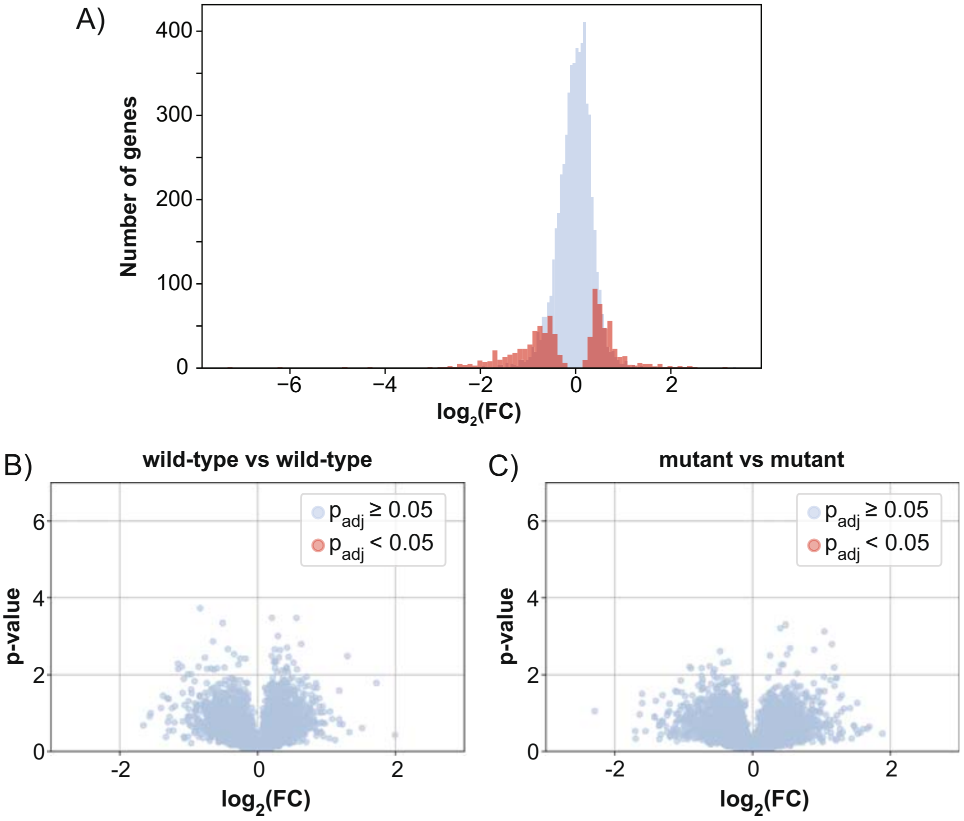

Figure EV1. Validity of the gene set that is identified as having differential fitness between the wild-type strain and the polarity mutant.

(**A**) Significant genes have a larger effect size. Histogram of the log_2-fold changes shown in Fig. 2. The plot shows that genes that are flagged to have a statistically significant difference between the two genetic backgrounds (red bars) typically have larger fold changes. (**B**, **C**) Comparing transposon mutagenesis libraries obtained from the same genetic background yields no significant differences in gene fitness. Volcano plots are shown for comparisons between wild-type and mutant datasets. The replicate datasets of each genetic background (6 in total) were split and compared 3 vs. 3. Statistical significance was determined with Welch's *t* test and corrected for multiple hypothesis testing with the Benjamini–Hochberg procedure (p_{adj}). For both our wild-type strain and our polarity mutant, no false positives are found at a significance threshold of $P_{adj} < 0.05$.

© The Author(s)

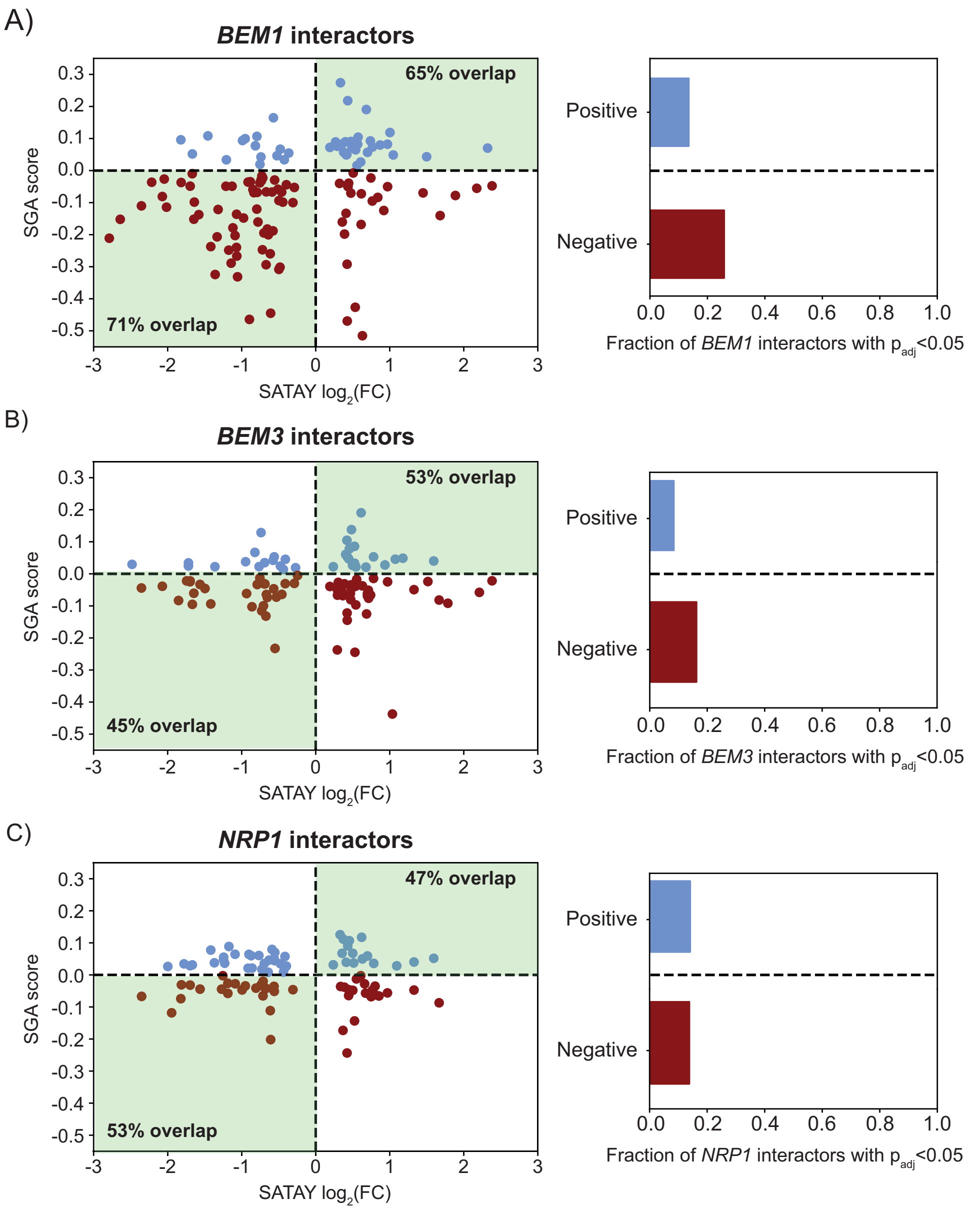

Figure EV2. Comparison of the genes with differential insertion tolerance found with SATAY to the genetic interactions identified by the SGA screen from Costanzo et al (2016).

Scatterplots illustrate the SGA scores plotted against the log-fold changes in gene tolerance for genes that both exhibit differential insertion tolerance in our SATAY screen and are identified as genetic interactors of (**A**) *BEM1*, (**B**) *BEM3*, or (**C**) *NRP1* by the SGA screen. Positive interactors (SGA score >0) are shown in blue, while negative interactors (SGA score <0) are shown in red. The percentage of genes with matching signs between the SGA score and $\log_2$ (FC) is displayed within each scatterplot (green-shaded area). The panels to the right of each scatterplot indicate the fraction of positive and negative genetic interactors from the SGA screen that were categorized as having differential insertion tolerance by the SATAY screen.

 © The Author(s)

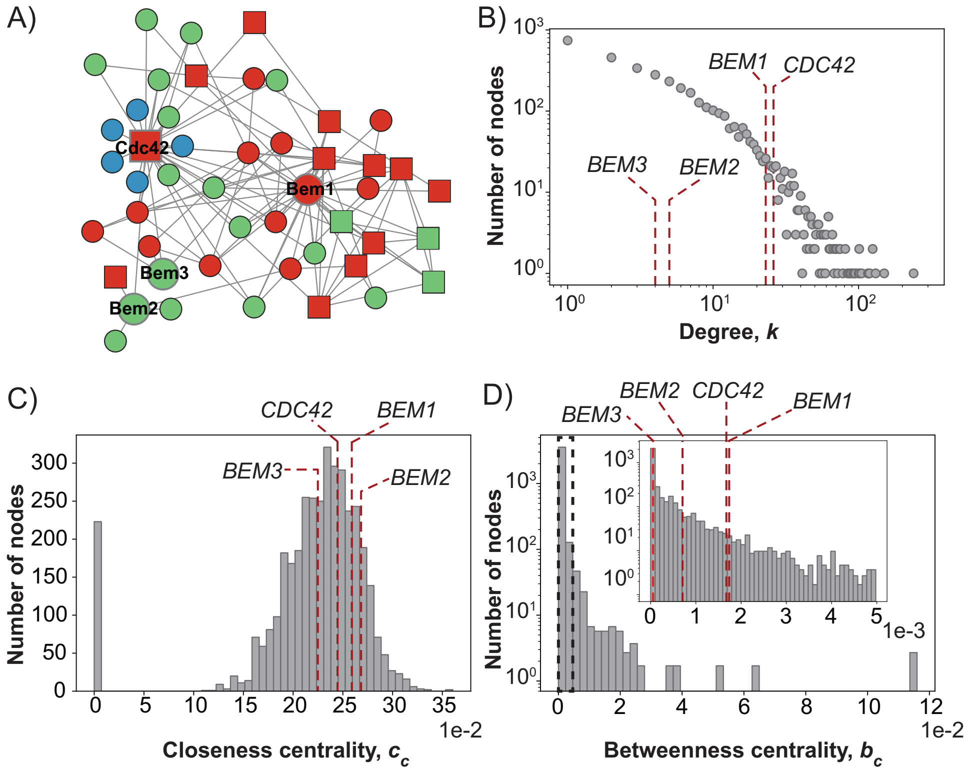

Figure EV3. Properties of the constructed protein-protein interaction network.

(**A**) Sub-graph of the protein-protein interaction network for Cdc42, Bem1, Bem2 and Bem3 and their first neighbors. Nodes are colored according to their degree k in the complete PPI network (Fig. 3A). Blue: $k \leq 3$, green: $4 \leq k \leq 10$, red: $k > 10$. Proteins that are essential according to the SGD database are shown as squares, non-essential proteins are shown as circles. (**B**) The degree centrality of the nodes in the complete PPI network. The degree distribution shows the typical sub-linearity of PPI networks in biology when plotted on a log-log scale. (**C**) The closeness centrality distribution of the PPI network. (**D**) the betweenness centrality distribution of the PPI network. The inset presents a zoomed-in view of the area outlined by the black dashed square. In (**C**, **D**), the degree values are indicated for two polarity proteins that have a strong negative effect on fitness when deleted (Bem1 and Cdc42) and for two polarity proteins that have a moderate to weak negative effect (Bem3 and Bem2). With respect to degree and betweenness, proteins with a similar gene fitness lie in proximity of each other on the distributions. For the closeness distribution, we find no relation with gene fitness.

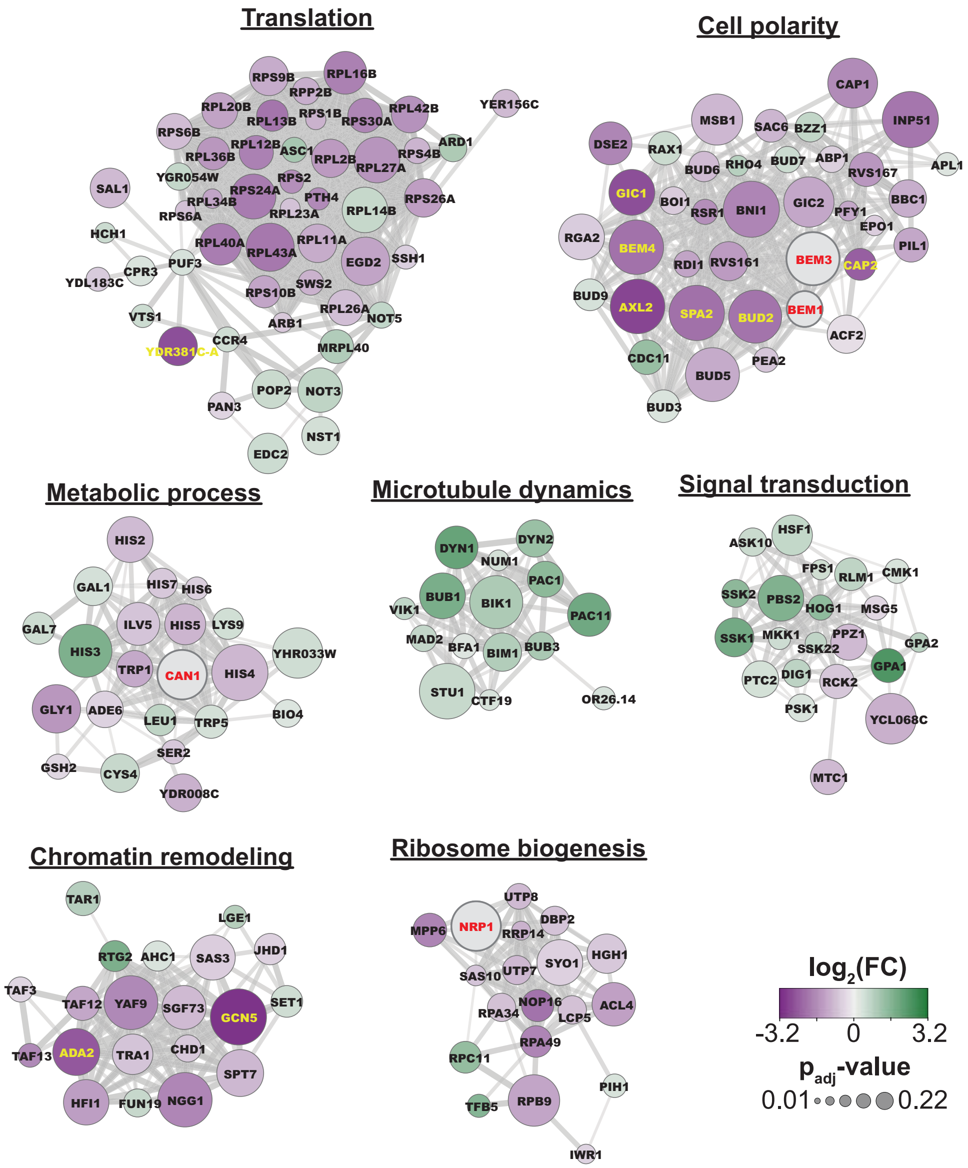

Figure EV4. Zoom-in of the seven largest clusters identified in our functional association network by the Markov clustering algorithm.

The biological process gene ontology enrichment is shown above each cluster.

© The Author(s)

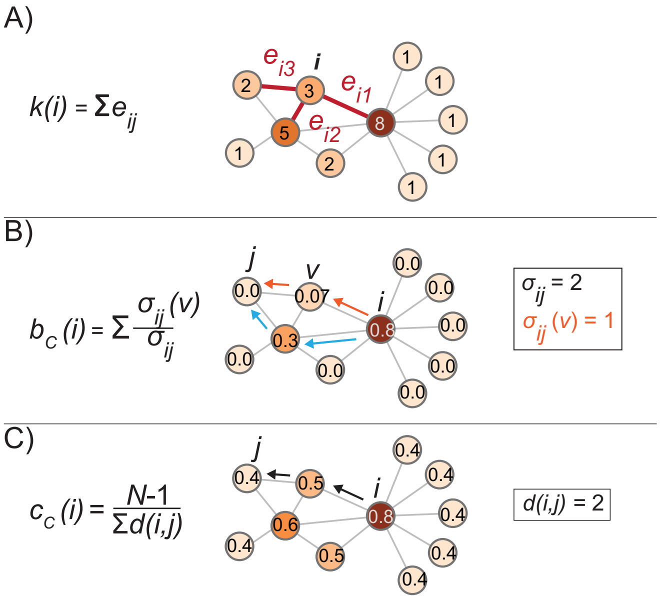

Figure EV5. A visual representation illustrating how the different centrality measures are calculated.

(**A**) The degree centrality k is determined by counting the number of edges e_{ij} a node has connecting it to other nodes in the network. The example graph shows a node i with a degree of three. (**B**) The betweenness centrality b_c provides a measure for the importance of a node for the information flow in the network based on the number of shortest paths that pass through that node. In the example there are two shortest paths σ_{ij} from node i to node j, but only one of these paths passes through node v. (**C**) The closeness centrality reflects the distance of a node to all other N nodes in the network based on the average shortest path $d(i, j)$. The example graph shows a node i with a shortest path length of two to node j.

© The Author(s)
